# How adolescents perceive their communities: a qualitative study that explores the relationship between health and the physical environment

**DOI:** 10.1186/1471-2458-14-349

**Published:** 2014-04-12

**Authors:** Kristin Mmari, Hannah Lantos, Heena Brahmbhatt, Sinead Delany-Moretlwe, Chaohua Lou, Rajib Acharya, Adesola Sangowawa

**Affiliations:** 1 Department of Population, Family, and Reproductive Health, Johns Hopkins Bloomberg School of Public Health, 615 N. Wolfe Street, Baltimore, MD 21205, USA; 2 Witts Reproductive Health and HIV Institute, University of Witwatersand, Johannesburg, South Africa; 3 Shanghai Institute of Planned Parenthood Research, Shanghai, China; 4 Population Council, New Delhi, India; 5 College of Medicine, Institute of Child Health, University College Hospital, Ibadan, Nigeria

## Abstract

**Background:**

The Well-Being of Adolescents in Vulnerable Environments (WAVE) study was conducted among adolescents aged 15-19 years in Baltimore, Ibadan, Johannesburg, New Delhi, and Shanghai to examine perceived factors related to their health. A preliminary analysis of the data, unexpectedly, revealed that the influence of the physical environment on adolescent health was a dominant theme across every site examined. To explore this further, this paper analyzed the specific components of the physical environment that were perceived to influence health, and how they contributed to various health outcomes across sites.

**Methods:**

Researchers in each site conducted in-depth interviews among adolescents; community mapping and focus groups among adolescents; a Photovoice methodology, in which adolescents were trained in photography and took photos of the meaning of ‘health’ in their communities; and key informant interviews among adults who work with young people. A total 529 participants from across the sites were included in the analysis.

**Results:**

Findings showed that while there was surprising uniformity in how adolescents characterized their physical environment, perceived health outcomes related to the physical environment varied by site and gender. In Baltimore and Johannesburg, vacant homes and the lack of recreation facilities were perceived to impact on sexual and reproductive health problems for girls, while among boys they contributed to drugs and violence. In Shanghai, New Delhi, and Ibadan, garbage and trash observed in their communities were perceived to have a higher impact on infectious and chronic diseases.

**Conclusions:**

As the world continues to urbanize, our study points to a strong need to examine how the physical aspects of a living environment contribute to the health of adolescents. Specific aspects, such as housing, safety, garbage, and recreational spaces must all be examined as possible pathways for making improvements to health of adolescents, particularly among those living in poor urban environments.

## Background

More than half of the world’s population live in cities, and by 2030, three-quarters of the world’s population will be living in cities [1]. Most of this growth will occur in low and middle-income countries, thereby expanding the growth of inner city and slum communities [2]. Additionally, much of the growth is the consequence of rural to urban migration with youth under 25 being the largest portion of such migrants [3].

Research has shown that living in an urban environment influences every aspect of health and well-being, including the choices people make about food, employment, housing, and sexual partners. However, despite the fact that a disproportionate number of urban migrants are young people between the ages of 16 and 24 years, we know very little about how the urban environment influences the health of adolescents, especially in low resource settings. An additional risk factor associated with urban migration is that while most young people migrate to urban areas in search of educational or economic opportunities, they often end up residing in poor, inner-city neighborhoods with fewer opportunities to access and utilize positive resources for maintaining health [3]. In fact, there is such inequity in many of these settings, that urban poor adolescents may be experiencing an “urban health penalty” that makes them worse off than their rural peers.

To understand how adolescents in these urban poor communities perceive their health and the factors that contribute to their health, the Well-Being of Adolescents in Vulnerable Environments (WAVE) study was conducted among adolescents aged 15-19 years in five cities around the world: Baltimore, Ibadan, Johannesburg, New Delhi, and Shanghai. A preliminary analysis of the qualitative data, unexpectedly, revealed that the influence of the physical environment on adolescent health was a dominant theme across every site examined [4]. Across all study sites adolescents characterized their communities as ‘dirty’, ‘overcrowded’, ‘dangerous’, and ‘polluted’, and described multiple ways in which the physical environment contributed to their poor health. While the influence of the physical environment on health is not a new concept, what was surprising was how pervasive this association was among adolescents independent of location. The primary objectives for this paper, therefore, were to examine how young people describe the physical environment, explore how adolescents perceive the physical environment to influence their health, and examine how these perceptions vary across site.

While the physical environment has been defined to include the built structures, the air and water, the indoor and outdoor noise, and the parkland inside and surrounding the city [5], for the present analysis youth were at liberty to define their physical environment as they chose. Since young people may view the physical environment (and built environment) differently than adults, this study also represents a first attempt at incorporating their perceptions about the physical environment and identifying specific aspects of the physical environment that may carry more meaningful implications for the health and well-being of young people in particular.

## Methods

### Research design

The Well-being of Adolescents in Vulnerable Environments (WAVE) study was launched in the summer of 2011 to: 1) explore adolescents’ perceived health and their top health challenges; 2) describe the factors within their urban communities which were perceived to be related to their health and health seeking behaviors. The study was conducted in the following five urban sites across the globe in order to gain a cross-cultural perspective about how adolescent health and health seeking behaviors may differ across cities: Baltimore, Johannesburg, Ibadan, Shanghai, and New Delhi. The choice of these cities was largely influenced by existing research relationships between the research team but in addition they represent major urban areas with sizable economically disadvantaged populations. For each city, the local research team selected a specific geographical area as the study site, based primarily on high poverty. Table 1 summarizes the characteristics of each site.

**Table 1:** Selected characteristics of study communities

**City**	**Selected community**	**Characteristics**
Baltimore	East Baltimore	High prevalence of low income residents near the Johns Hopkins medical campus; majority of residents are African American
Shanghai	Sub-district	A suburban area located in the northwest of Shanghai, the size is 18.8 square kilometers with about 200,000 inhabitants and over half of inhabitants are migrants
Johannesburg	Hillbrow	Densely populated inner city area (size is about 1 square kilometer with approximately 100,000 inhabitants); community is characterized by high levels of poverty and crime; made up of local Johannesburg residents and immigrants
Ibadan	Ibadan North Local Government Area, Oyo state	Third largest city in Nigeria; capital of Oyo state – within the city, a poor ‘inner city’ community was selected; predominant ethnic group was Yoruba
New Delhi	A slum in one of Delhi’s four districts (South Delhi) bordering the state of Haryana	Large slum community inhabited by migrant families from different parts of the country, overwhelmingly poor, lacking basic facilities, such as sanitation and water

### Sampling

A purposive sampling frame was used in each city to select adolescents and adults who worked with adolescents. Male and female adolescents aged 15-19 years who lived within the selected cities, as well as representatives of the identified youth-serving organizations were recruited and were contacted either in person, by telephone, or a mailed letter explaining the purpose of the study, the data collection activities, and the risks and benefits of study participation. In some sites, key informants were also asked for the contact information of adolescents who could be recruited to participate in the study. Since these community agencies provided a broad array of services, it assured access to a diverse adolescent sample frame from which to recruit. Across all sites, adolescents were only recruited for one type of research methodology to ensure that each data collection method (i.e., in-depth interviews, Photovoice, and community mapping/focus groups) had different adolescent participants, allowing for a greater breadth of adolescent perspectives.

### Data collection

Data were collected in the summer of 2011 using identical research protocols across the five study sites: key informant interviews among representatives from schools, places of worship, and youth-serving organizations; in-depth interviews among adolescents; community mapping and focus groups among adolescents; and a Photovoice exercise among adolescents. Each of the methods was decided upon through a series of discussions among researchers across the sites. Given that we wanted to include different methods that would offer us the ability to triangulate our findings, we decided that community maps/focus groups, in-depth interviews, and Photovoice would be able to provide this opportunity for us.

To ensure that researchers followed the same research protocol across sites, trainings were developed by the lead qualitative investigator (KM) through the use of video clips and power-point presentations, and role-play exercises that used the research instruments. Consent procedures were also standardized across sites. Written adult consent was obtained from adolescent participants aged 18 years and over in every site except Shanghai, where the age of majority was 16 years. For adolescents younger than 18 years (or 16 years in Shanghai), a combined written parental/guardian consent and child assent form was signed. A parent or guardian could include anyone who had legal authority over the child, which in some cases meant directors of homeless shelters or the Social Services Administration for foster children in Baltimore. All research protocols were approved by the Johns Hopkins Bloomberg School of Public Health IRB, and subsequently at each site’s human ethics review committee, which included the University of the Witwatersrand Human Research Ethics Committee (Medical) in Johannesburg, the University of Ibadan/University College Hospital Ethics Committee, the Medical Ethical Committee at the Shanghai Institute of Planned Parenthood Research, and the Population Council IRB in New York City and the Futures Group Institutional Ethics Committee in New Delhi.

#### Key informant interviews

Representatives from approximately 20 youth-serving organizations were asked to participate in the key informant interviews. These participants included teachers, religious workers, social workers, directors, and youth workers. In Shanghai, researchers purposively selected adults who worked with migrant youth in the study site. The primary purpose of these interviews was to ask adults who worked with youth in the selected sites about what they considered to be the biggest health challenges faced by adolescents, and the factors that contributed to these health challenges. Each interview lasted approximately 30-60 minutes and was audio-recorded.

#### In-depth interviews

At each site, approximately 20 adolescents aged 15-19 years were asked to participate in the in-depth interviews. Emphasis was placed on ensuring that there was a balance in gender and age distribution (i.e., 5 males, aged 15-17 years; 5 males, aged 18-19 years; 5 females, aged 15-17 years; 5 females, aged 18-19 years). The interviews were conducted to explore how adolescents perceived their health, what they considered as their main health challenges and the factors that contributed to these challenges, and how they sought help for their health problems. Similar to the key informant interviews, the in-depth interviews lasted approximately 30-60 minutes and were audio-recorded.

#### Community mapping and focus groups

Approximately eight community mapping/focus groups were conducted at each site. Similar to the in-depth interviews, groups were divided by age and gender: males, aged 15-17 years (2 groups); males, aged 18-19 years (2 groups); females, aged 15-17 years (2 groups); females, aged 18-19 years (2 groups). The methodology started out with the community mapping exercise, followed by the focus group to collect additional information about comments that were mentioned during the community mapping.

**Figure 1:**
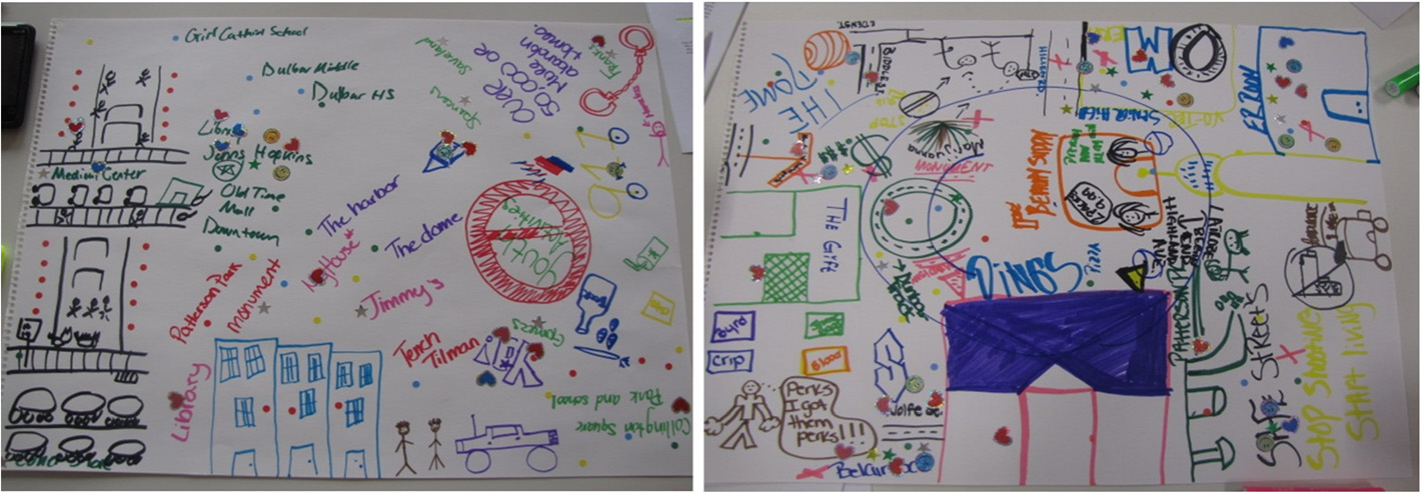
Community map illustrations.

In the community mapping exercise, the group of adolescents was provided with flipchart paper, colored markers, and stickers representing different aspects of the community, and was asked to first draw a map of what they perceived to be their ‘community’. Stickers and markers were then used on their maps to represent areas where they liked to ‘hang out’ in, that they thought were ‘safe’ and ‘unsafe’, where bars, schools, recreational areas were and where they thought they could get ‘help’ for various health issues, such as violence, mental health, or sexual and reproductive health problems. Focus groups were then conducted to gain further insight on the community context and how it contributed to their health. All discussions during both the community mapping exercise and the focus groups were audio-recorded, and photos of the all of community maps were taken for further analysis. Figure 1 illustrates examples of these community maps.

#### Photovoice methodology

Ten adolescents (5 male and 5 female) were recruited at each site to participate in a week-long Photovoice exercise [6]. Adolescents were trained in the art of photography and, for a week, took photographs that captured what they believed was the meaning of health in their community. At each site, the training was co-facilitated by either a professional photojournalist or an experienced photographer. After adolescents took photos, they selected the photos they felt best represented “the meaning of health” and gave each photo a caption that they felt explained their selected photos; the development of which was aided by group discussions.

### Analysis

All recordings were transcribed verbatim, translated into English, and uploaded into Atlas.ti (Scientific Software, Berlin; version 7). At each site that conducted translations, at least two research assistants were involved in translating the interviews. Additionally, the local PI at each site also conducted random ‘quality checks’ of the translations to ensure that the research assistants were translating appropriately. This was done by comparing the English and the local language transcriptions side by side. Once all the translated transcripts were uploaded in Atlas.ti, an inductive content analysis approach [7] was used for the analysis, where the qualitative investigators from each site began by reading each transcript to get a sense of the primary themes that were emerging. An initial core set of codes was developed by the lead qualitative investigator and then shared with the qualitative investigators across the sites.

As more transcripts were read across each of the sites, the core set of codes was revised until a final set of codes was achieved. This core set of codes was used for the initial coding schemes across all five sites; within each site, additional sub-codes were created that were site specific. At each site, two researchers coded the transcripts and the photos from the Photovoice exercise using an incremental, step-wise process, which first involved applying the core set of codes and then developing more analytical sub-codes. At each of these steps, a comparison of the codes within each coding team revealed if there were coder discrepancies; any discrepancies that did occur were discussed with the lead qualitative investigator from that site until consensus could be achieved. Coding concluded when all the data was assigned to a code, and saturation was achieved [8]. To compare the codes across sites for this analysis, matrices of the key codes were created that related to the physical environment and perceived health challenges.

### Trustworthiness of the data

A number of techniques were used to ensure trustworthiness of the findings [9-11]. As stated previously, researchers at each site participated in the same training and practiced using the actual data collection instruments to reduce potential researcher bias. We also triangulated the findings across methods to confirm emerging themes (i.e., themes that had emerged in the Photovoice exercise were also echoed in the in-depth interviews and community mapping/focus groups). In addition, in sites that had translations conducted, specific quality checks of the translations were conducted by the site PI to ensure English translation matched with the local language. Finally, consistent with the peer debriefing strategy [11], the initial core set of codes and set of preliminary findings were presented and discussed with members of the entire research team in an effort to verify both the coding structure and the interpretation of the findings across sites. Each of these techniques, as well as our methodology used for this study, abides with the RATS guidelines for qualitative research [12].

## Results

### How do young people describe the physical environment?

When youth were asked to describe their communities and the meaning of health in their communities, aspects of the physical environment were by far the most commonly described. In particular, there were five primary aspects of the physical environment that were mentioned as having an influence over their health: 1) the perception of safety; 2) dirt and pollution; 3) housing; 4) recreational spaces; and 5) water and electricity (infrastructure). Among these five aspects, the perception of safety was the most dominant theme, which was discussed frequently among adolescents across all five sites.

#### The perception of safety

In both Baltimore and Johannesburg, there were many youth who felt that there was actually no place that they felt safe – even in their own homes.

… The thing is that where we stay you will hear a person screaming from being beaten up in the middle of the night and there are also break-ins

*Interviewer: Don’t you have security guards in your complex?*

We do have them but people come in as they please

*Interviewer: Is there anything that’s been done about that problem?*

*The caretaker has been told about the problem and he changed the security company and is now using Bad Boyz but I still don’t feel safe.* (IDI, 19 year old female, Johannesburg)

*There ain't nowhere to be safe, tell you the truth. All I'm saying is it's not even safe to even walk around by yourself at a certain time, even though you don't have a curfew. It's not. Three years I got banked [beat up] so many times it doesn't make no damn sense. I got banked too many times, even walking late at night, 3, four o'clock in the morning. Just walking anywhere.* (Photovoice, male between the ages of 15 and 19 years old, Baltimore)

In other sites, adolescents mentioned that the only place they felt safe was inside their home; although in some respects, several of the adolescents were still not fully convinced that their homes would provide them with complete safety:

*No, there is nowhere that is safe except inside your house, inside your room. Even your room is not safe; it is only God that will help us.* (IDI, 18 year old female, Ibadan).

When asked about the reasons why youth felt unsafe in their communities, many youth mentioned that being in areas where there was a high concentration of people or a lot of noise made them feel unsafe. In Baltimore and in Johannesburg, several of the adolescents who were interviewed in community mapping exercises and in-depth interviews said that vacant homes or buildings were considered ‘unsafe’, as they were places where crime often took place. In Shanghai and Ibadan, adolescents also mentioned being unsafe in their communities because of the high volume of traffic and the risk for getting in accidents. Additionally, in Shanghai, many adolescents described how the lack of street lights made them feel unsafe at night.

*Interviewer: what makes you feel unsafe?*

*Because there are few lights on the park, and it’s very dark at night. It’s scary if I’m at the park alone at night, because there may be many villains.* (focus group, female between the ages of 15 and 17 years old, Shanghai).

Interestingly, in both New Delhi and Johannesburg, there were several specific places in their communities where girls in particular felt unsafe. Two different key informants in New Delhi suggested that there were areas where girls were at a high risk of assault or that *“girls were not safe in the buses; there are even teasers who tease them”.* Given the recent news events of girls getting gang raped on buses in India [13,14], stories of girls being unsafe on buses may seem particularly relevant and striking.

#### Dirt and pollution

Another common theme was that the environment was characterized by dirt, garbage, and pollution in all of the study communities. In Baltimore, adolescents frequently described the heaping piles of trash right in front of where they lived, which often attracted rats and other rodents; and this was captured in pictures taken in almost all the sites.

*It's dirty. The people, they don't put their trash in the trashcans. They put it in the alley right there. And the people, when they come in the morning to get the trash, what they don't know is they're making it worse because you're bringing more rats. And when the wind blows, when it rains, or anything, and the kids, they'll come and they'll kick the trash or they'll throw the trash. It's nasty! That's why you can't even sit out on the step at nighttime. It just be dirty.* (Photovoice, female between the ages of 15 and 19 years old, Baltimore)

Similarly, in Shanghai, youth frequently described the trash and garbage that surrounded their communities and even parks. They also further described the ‘dirt’ from the industrial pollution being emitted from the factories in the area.

There is a tofu factory and the river is full of dirty discharge from this factory.

*Some people would remove the dirty things in the river. The river was once very clean, but it became very dirty after some factories opened. There are many factories near the river, such as furniture factory and food factory, which discharge something really dirty. Some people would remove the garbage by boat in the morning, but it’s still dirty.* (focus group, female between the ages of 18 and 19 years old, Shanghai)

In New Delhi, adolescents and key informants said that the garbage and dirt filled the air and water with filth, impacting recreation as well's health.

*…There is so much of dirt here and smell keeps on coming from everywhere, the dirty water is found lying on the roads, the dirt is spread everywhere, the drains are full of filth. If they clean the drains after two or four months so the garbage is found lying there for many days. The atmosphere over here is very bad and because of this dirt it affects badly on the health and the diseases also spread. There is no place available here for playing.* (Key Informant (no age or gender provided), New Delhi)

**Figure 2:**
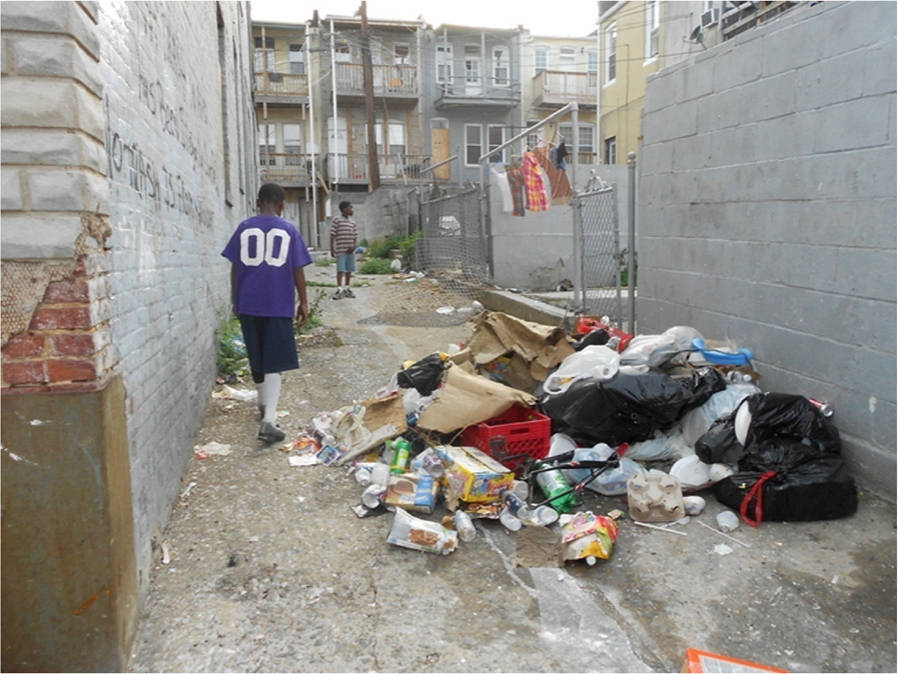
**In order to have a healthy community you have to have neighbors who are willing to do something about it.** By: 17 year old Baltimore Photovoice participant.

**Figure 3:**
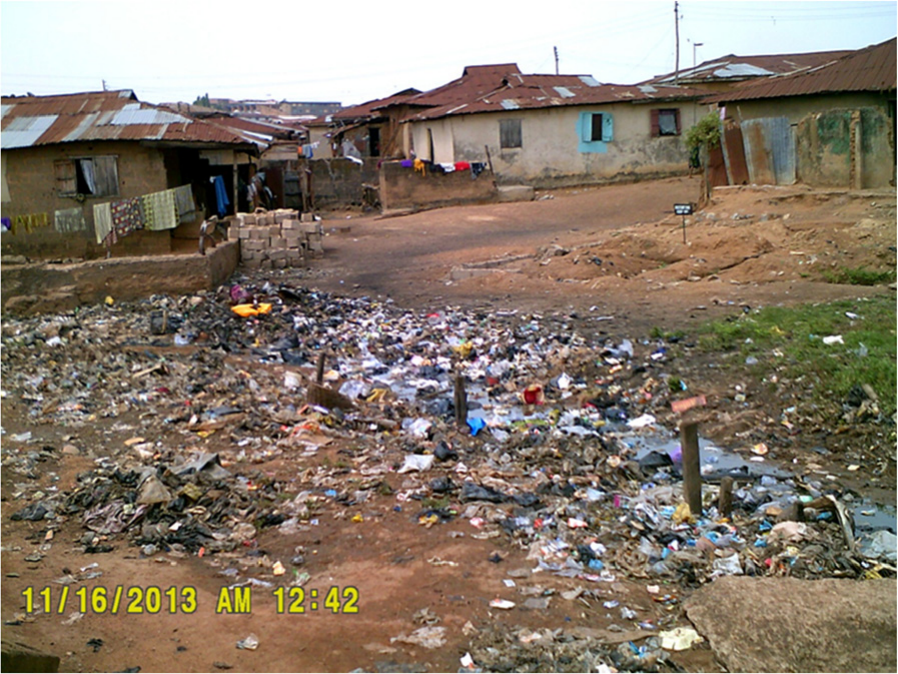
**People living around this very dirty environment may contact diseases and they can’t have good, clean fresh air to breath.** By: 18 year old Ibadan Photovoice participant.

Photos taken as part of the Photovoice exercise further echoed this theme of garbage and trash filling their communities (Figures 2 and 3).

#### Housing

Across the sites, a third major aspect of the physical environment that was described in great detail and with equal concern was the housing. In Baltimore, this was primarily described in terms of both vacant and decaying/boarded-up houses.

*But they can't fix up the houses if the drug boys in there keeping stashes and stuff…Yeah, that's why they board - when people move, they board up the houses now so nobody can break in. Every time they board it up, it's getting unboarded. Like this house on the corner where I live at, it's going to an apartment now, every time they put a glass window up there three days later its busted open.* (Photovoice, male between the ages of 15 and 19 years old, Baltimore)

In other sites, such as Johannesburg, Shanghai, and Ibadan, it was often described by overcrowded housing.

*What I don’t like is how overcrowded it is. It’s a place with lots of people. It’s overcrowded; when you turn on one side, it’s full and when you turn another, it’s full.* (IDI, 18 year old female, Johannesburg)

*We have houses there, but they are too crowded together and overpopulated.* (focus group, female between the ages of 15 and 17 years old, Ibadan).

In a focus group of females between the ages of 15 and 17 years old in Shanghai, one young women described how most of the homes where migrants live are small rented homes, many of which have no windows and can be shared by more than one family, which another participant in the same focus group said ‘*often leads to chaos’*.

#### Infrastructure

Another aspect of the physical environment that was repeatedly mentioned by participants in New Delhi and Ibadan was the infrastructure of the city, especially in terms of the lack of electricity and water as well as the damaged drainage systems. In New Delhi, one male adolescent said *“There are no facilities from government side like for water, there is just one tank and everyone drinks water from there”.* In Ibadan, four adolescent females between the ages of 15 and 17 years also commented on the lack of basic services – from electricity to water to sewage – in their specific community. One young woman said, “*There is no toilet there and also there is irregular supply of electricity”*.

Interestingly, in New Delhi, many of the comments about access to water linked back to perceptions of a lack of general cleanliness in the community. One young boy in the Photovoice group said:

Here the water supply pump is installed and just besides it there is a dirty water drain and one boy is standing here and taking bath. His foot can slip and he can also fell into the well. And the clothes are drying on the pump of water supply and the water can get polluted from this and we could suffer from diseases.

#### Recreational spaces

Finally, in all of the sites, youth described the quality of the places that they can use for hanging out and recreation. In most sites, adolescents felt there were limited places where they could feel safe to socialize and engage in recreational activities. In Baltimore, this included parks and playgrounds in their community.

*There is no youth activities, period. Like my nephews, they are wanting at least, “Can I go outside, can I go outside?” And do what? No, you're not going outside. Now, if you had a specific thing to do? Yes, then can go outside but you're not going outside with nothing to do. No activities and nobody supervising you. No. The playground isn't safe. You can't even push a little baby on the swing.* (focus group, female between the ages of 18 and 19 years old, Baltimore)

Interestingly, in Shanghai, the majority of adolescents felt that there were few ‘healthy’ public recreation spaces where adolescents could get access to sports facilities or green spaces.

*There are no sports equipments, no facilities. There is no place to play badminton. The basketball court does not have basket. A lot of young people don’t have place to play. For girls like us, if there are blocks of lawn to be laid down, reading with our pals, with music surrounded, it would be very well. But the problems are, first there is no person to be with, second there is no such a place.* (Photovoice, male between the ages of 15 and 19 years old, Shanghai)

Rather, they felt that there were ‘too many’ recreational spaces that were not promoting healthy behaviors, such as internet cafés where boys get access to violent video games and pornography.

*They should close most of the internet cafes, and KTVs, and the footbath room. These recreational places could be opened, but not so many. And they should be under strict control. The Internet cafes let youth to play games, and some of the games are a little violent. It makes you addicted. And stay there overnight.* (focus group, male between the ages of 15 and 17 years old, Shanghai)

In New Delhi, most of the discussion surrounding recreational spaces centered on the limited options for girls. Repeatedly, boys were said to have far more freedom within the community, and could access more places to meet peers, such as internet cafes.

### What are the various ways in which the physical environment influences adolescent health?

For each of the physical environment components that were discussed among adolescents, there were specific health consequences that youth discussed. In particular, there were four outcomes that were frequently mentioned as being associated to the physical environment: exposure to chronic and infectious diseases, sexual and reproductive health vulnerability, access and use of drugs, and experiences of violence. Interestingly, the health consequences of the physical environment varied by setting

#### Chronic and infectious diseases

Dirt and pollution as well as infrastructure concerns were related to chronic and infectious diseases. Adolescents, especially from New Delhi, Baltimore, and Ibadan remarked at how these conditions, especially within a person’s house, can spread disease quickly.

*… Here the dirt is too much. Here people clean their house but what would happen from it if there will be dirt outside, so dengue mosquitoes will breed, they come inside the house and bite. The disease also spread, so one should keep cleanliness outside the house and inside too, you can see that how dirty is it.* (IDI, 17 year old female, New Delhi)

*There is no toilet there and also there is irregular supply of electricity… Like dirty water that brings mosquitoes into the house… Dumping of refuse; provision of incinerators or refuse grounds far away from human habitation prevents air pollution and ill-health.* (focus group, female between the ages of 15 and 17 years old, Ibadan)

*The worst thing I hate if my mother cooks something and I go to bed and wake up tomorrow and see the food on the table. It gets on my nerves, because that's a big health issue. It's sitting out overnight. We've got my niece in the house. Now she climbs up on stuff and gets into stuff. We keep putting food out there…She's going to eat it. She might get sick. Roaches are going to start coming, and that's a big health issue.* (Photovoice, female between the ages of 15 and 19 years old, Baltimore)

This dirt combined with a lack of infrastructure in the homes put youth in many of these sites into situations where they felt they were more likely to be exposed to diseases transmitted by mosquitoes or other bacteria. In Johannesburg, one key informant mentioned that the overcrowded living spaces were perfect “breeding grounds for tuberculosis”, which was mentioned by several adolescents as being a commonly spread disease.

In Shanghai, interestingly, adolescents primarily related the fumes from the factories or the pollution from the rivers as being the main contributing factors to chronic disease.

*One example is the river out of our neighborhood is very stinky in the summer. My father told me, when he first came to Shanghai, the river was very clean, and there were fishes swimming. But after these years fast development of the city, there are no longer fishes. I doubt if a toad would like to come. The environment is so heavily polluted that we don’t even want to walk by….The air around must not be very fresh, and must be very toxic.* (Photovoice, female between the ages of 15 and 19 years old, Shanghai)

#### Sexual and reproductive health

In Baltimore and Johannesburg, adolescents linked sexual and reproductive health problems explicitly to vacant homes and the lack of recreational activities.

*So, these abandoned buildings are also a problem to the community because of crime, drugs, rape and prostitution all take place there.* (Key informant (no age or gender provided), Johannesburg)

*We don't interact with each other enough. Every time you turn around, you're standing in a guy's face or there's a guy you're messing with. So I think there needs to be more activities for women than for us to just go to school, come home, and lay on our bed. There's no activities for us and it causes us to reproduce a lot. They don't understand that.* (IDI, 18 year old female, Baltimore)

In Johannesburg, one key informant said it was the combination of both overcrowding and the lack of recreational activities available to young people that were inadvertently exposing children to all kinds of risk behaviors.

*… be it domestic violence, neighborhood violence, alcohol abuse, sexual abuse… It just becomes normalized into that behavioral repertoire. They [the children] internalize that, and then we find that children, as a result, re-enact a lot of what they have been exposed to*.

While these were issues for youth in Johannesburg and Baltimore, adolescents from Shanghai, Ibadan, and New Delhi did not link the physical environment with sexual risk.

#### Drugs and alcohol

With the exception of Shanghai, drug and alcohol use was mentioned as another health outcome that was perceived to be associated to their physical environment. In Baltimore, several adolescents and key informants associated the presence of vacant homes to drug access and exposure.

*And not only that, you've got people staying in the vacant houses. And so what is the health department doing? They're busy doing nothing. They are coming out, what are you doing? You've got drug needles on the ground.* (Photovoice, male between the ages of 15 and 19 years old, Baltimore)

One female Photovoice participant from Baltimore described a story where she was at risk from being killed by a fire that started by drug users in a vacant home near-by.

It was like a couple doors down and the junkies kept going in there and free-balling. And they started a big old fire that went from that house all the way down to mine. We were in there asleep. My sister, it was a good thing she woke up. I don't know. They free-balling all up in there.

While youth from New Delhi and Johannesburg discussed the exposure and availability of drugs or alcohol in their communities, they did not link them to vacant homes. Instead, they primarily discussed the availability of liquor stores and people selling drugs on streets as being the main contributing factors for drug use.

*…and I don’t like the fact that it’s full of foreign guys selling drugs and I hate the fact that they are selling drugs at almost every street corner and to them there is nothing wrong with it.* (IDI, 19 year old female, Johannesburg)

*This area is not safe for the educated children because in every street there are wine shops, the people take intoxicants and the outsiders also come to take intoxicants and when the children pass away from that place, so how bad impact it have on them* (Key Informant (no age or gender provided), New Delhi)

#### Experiences of violence

Just as youth above described a feeling of pervasive fear, many of the youth associated this lack of safety with actual experiences of violence. In Baltimore, Johannesburg, and, to a certain extent, New Delhi, the acts of violence primarily involved instances of assault, shooting, or harassment. In Baltimore, one young woman in a focus group of 15 to 16 year old females said, “*People run in their houses and they get shot.”* Several other adolescents in Baltimore who participated in the in-depth interviews and the Photovoice exercise supported this sentiment and described that violence was nearly everywhere in their community – from the playground, to the streets, to their homes.

*Yeah because I see [domestic violence] a lot. I really see it a lot. They do it in front of the kids nowadays. These days now, these days now these young men don't care. They do anything in front of their kids, outside in front of people, anywhere, it don't matter.* (IDI, 19 year old female, Baltimore)

*… My little sister, one day I took her to the park. She picked up a plastic bag in the park, and I'm trying to figure out why she was dragging the bag. The man was like, “Yo, yo, yo, don't let her touch that!” So I picked up the bag and looked inside the bag. There was a gun in the bag. If that thing would have been unlocked or something, she could have shot herself. That's dangerous.* (Photovoice, female between the ages of 15 and 19 years old, Baltimore)

In Johannesburg, there were a few girls who also felt unsafe in certain places of their community, primarily due to the higher concentration of immigrant and homeless groups.

*I don’t feel safe around Nigerian men…When they are crews and drinking alcohol and you pass they will just do anything they want to you. It’s not only the Nigerians who I don’t feel safe around. Also the homeless people, they will chase you. I was once chased by one of them coming from Spar in the morning and I had to get into another shop and I told them that there is a guy chasing me and he was standing outside laughing at me. *(focus group, female between the ages of 15 and 16 years old, Johannesburg)

Interestingly, in New Delhi, experiences of violence were primarily described in the context of alcohol consumption, in which men would become intoxicated and then harass girls who would walk by them, particularly at night. As illustrated by the quote below, many felt that if the liquor shops would shut down, there wouldn’t be as much violence.

*Intoxication is also one of the main challenges here. There are many liquor shops and if they will be closed, then many improvements could be made here. When the boys over here see that their elders are consuming intoxicants so they also start to consume intoxicants. The main challenge for the girls is teasing. The girls don’t used to consume intoxicants but because of intoxication they also have to face problems as the boys used to molest the girls since they are intoxicated. *(Key informant (no age or gender provided, New Delhi)

Others in New Delhi described certain locations in the community, whether it was the main road or near the canal, where girls were more likely to be harassed.

*They [boys] hold the hand of the girl, come in their way and pass lewd comments. All this happens with them…It happens here on the main road only. It happens on the main road and behind the toilets also. (Showing on the map) people of this area do it more often. They tease the girls. It happens near the canal also. *(focus group, male between the ages of 15 and 17 years old, New Delhi)

In Shanghai and Ibadan, there were very few respondents who brought up any personal experiences of violence. Instead, adolescents in these cities primarily discussed feeling at risk of having certain items stolen from them, such as cell phones or bicycles. In Shanghai, adolescents frequently pointed out specific locations where robbery and theft were more likely to occur:

*There are so many such things happened in the Qian Long Bridge area. There was someone robbed of necklace on the roadside. It always are two or three person, one is in front of you, one is behind you, and the other one grabs something from you, like your wallet. Or someone robs you of your bicycle when another one prevent you resisting. *(focus group, female between the ages of 18 and 19 years old, Shanghai)

## Discussion

This analysis explores how adolescents living in disadvantaged urban environments perceive their physical environment and the impact it has on their health. Despite the different geographic locations of the study sites, there was surprising uniformity in how adolescents characterized their physical environment. Safety, dirt, infrastructure, and recreation were themes that emerged repeatedly and across multiple sites.

Adolescents across the five sites perceived their communities as being unsafe and dirty; and for both males and females in Baltimore and Johannesburg the lack of safety extended into their own homes. Gender differences were also a consideration when youth discussed safety. Specifically, girls in New Delhi and Johannesburg felt more vulnerable in their communities compared with their male counterparts. Adolescents across sites also commented on the dirt, trash, and poor housing that existed in their communities.

Geographic differences, however, did emerge when youth described the infrastructure considerations and the lack of recreational activities. Adolescents in Baltimore and Shanghai, for example, rarely mentioned infrastructure factors as having a huge influence over their health while adolescents and key informants in both Ibadan and New Delhi described this aspect in much greater detail, especially in relation to the water drainage systems and lack of electricity. Similarly, adolescents in Baltimore, Shanghai, and Johannesburg described the lack of positive recreational activities as a major concern of their communities. Interestingly, in New Delhi, very few adolescents mentioned the lack of recreational activities at all; and when it was discussed, it was described in terms of females having less access to ‘hang out’ places in comparison to their male counterparts.

In contrast to the general consensus about aspects of the physical environment, there was more variation across sites as to what health outcomes youth perceived to be related to these environmental factors. For instance, in both Baltimore and Johannesburg, adolescents primarily associated sexual and reproductive health problems, drugs, and violence to vacant houses and the lack of recreational activities. In Shanghai, Ibadan, and New Delhi, chronic and infectious diseases were the primary health outcomes linked to the physical environment, although harassment among girls was also seen by respondents in New Delhi as being higher around liquor shops and in certain areas of the community.

Placing these findings within a broader historical perspective, it should not be surprising that adolescents in these disadvantaged urban communities perceived the physical environment as a key factor influencing their health. In fact, for decades researchers and policy makers have been aware of the fact that the environment affects health [15]. Historically, this was driven by the fact that urban dwellers were less healthy than their rural counterparts; as people began to live in large urban environments, water and air-borne infectious diseases began to create an “urban penalty” where urban residents had lower life expectancies than their rural peers. Public health departments and systems emerged as the mechanisms to address the health needs many of which emerged as a consequence of people living in close proximity to each other. Sewer systems, water treatment facilities, laws governing the emissions of air-born pollutants first developed in urban areas throughout Europe and the United States with the aim of trying to stop the spread of disease. With the success of such interventions the “penalty” of urban residence was moderated [3].

In the public health context of today, there is emerging evidence that the modern city has many of the old as well as heretofore unknown public health challenges. The rapid increase in urbanization during the past few decades has coincided with a period of economic stagnation in many low and middle-income countries; and the result is a proliferation of impoverished slum settlements and inner city neighborhoods -- characterized by decaying physical environments, differential quality of education, targeted alcohol and tobacco advertising, and high rates of crime and violence [16]. Concurrent with this rise of vast, impoverished urban communities across the world, there is a growing and renewed interest in understanding how specific aspects of poor urban environments are affecting the health of its residents. Galea and Vlahov, for example, proposed an urban health framework to categorize the theories and factors that have so far emerged to explain how urban communities influence the health of its residents [5]. Notably, the physical environment is one of the framework’s three central components; the other two being the social environment and the availability and access to health and social services.

When we examine the research that has so far been conducted on the physical environment and its relationship to health, two observations can be noted. First, within the field of adolescent health, the physical environment has primarily been studied by measuring a community’s ‘walkability’, access to healthy food, as well as the amount of green space in relation to adolescents’ physical activity, BMI, and obesity [17-25]. Only one study in the adolescent health literature was identified that examined the physical environment in relation to health outcomes other than physical activity and obesity, and that study was conducted in South Africa [26]. Accordingly, the authors of that study examined the relationship between the physical environment and sexual risk behaviors among adolescents in urban Cape Town, South Africa. The authors’ measure of the physical environment included access to: 1) water; 2) sanitation; 3) electricity; and 4) housing quality. Notably, they found that with this measure, the physical environment was significantly associated with sexual risk taking among adolescents: youth who scored high on the physical environment scale (meaning they had higher access to services) were much more likely to use a condom at last sex and have fewer sexual partners compared to those with lower scores, controlling for sociodemographic variables [26].

The second observation about the current body of literature on the interrelationships between physical environment and health is that there is no one consistent measure of the physical environment. For example, Miranda et al. created a measure that included the physical infrastructure (e.g., buildings, roads, and lighting) and outdoor spaces (e.g., parks and urban design) of a given community, and compared those measures with the urban policies that might affect those aspects [24]. Another recent paper attempted to create a built environment index by combining observed neighborhood structures as well as crime and tax data [27]. Duncan et al, meanwhile, used software to develop a measure of the places to which adolescents could walk [19]. They included “recreational open space per square kilometer, parks per square kilometer, bus stops per square kilometer, subway stops per square kilometer, total retail walking destinations (e.g. clothing stores, pharmacy/drug stores, bookstores) per square kilometer, total service walking destinations (e.g. post offices, banks, credit unions) per square kilometer and total cultural/educational walking destinations (e.g. movie theaters, schools, libraries) per square kilometer”.

When our findings are compared with the extant literature we see, first of all, that not one adolescent in any study site mentioned obesity or physical activity in relation to their physical environment. While the current body of literature does provide evidence that the physical environment is associated with obesity and physical activity, our study suggests that in disadvantaged urban communities, there may be a need to examine the relationship between the physical environment and health using a much broader perspective than currently exists. This perspective should not only expand on the aspects of the physical environment beyond ‘walkability’ measures, but also focus on how such attributes may directly, or indirectly, be associated with adolescent health outcomes. In particular, given that there was only one study found on the relationship between the physical environment and sexual and reproductive health, there is need for more research that examines other attributes of the physical environment, especially since our findings suggest that vacant buildings and the lack of recreation spaces may be important contributors towards sexual risk taking among adolescents.

Secondly, one of the most important findings, the perceived lack of safety, has rarely been incorporated as an item of physical environment measures. This is not surprising given that perceived safety is, in of itself, not an objectifiable aspect of the physical environment. Researchers utilizing an urban health framework might also conceptualize perceived safety as an aspect of the social instead of the physical environment. The social environment, for example, has been defined at the individual level in terms of social networks and at the neighborhood level in terms of social cohesion and social capital. However, our findings suggest that perceived safety is intimately linked with other aspects of the physical environment. While some would argue that ‘trust’, which is an aspect of social cohesion, is closely aligned with perceived safety, in our study, adolescents described safety in terms of specific places or locations – irrespective of the social relations within their communities. Specifically, vacant homes, unlit streets, and noisy or overcrowded areas were frequently mentioned ‘places’ that made them feel unsafe. Future research should examine perceived safety especially in terms of how it relates to adolescent health and as a dimension of the physical environment.

## Conclusions

As the world continues to urbanize, our study points to a strong need to examine how the physical aspects of a living environment contribute to the health of adolescents. Specific aspects, such as housing, safety, garbage, and recreational spaces must all be examined as possible pathways for making improvements to health of adolescents.

## Competing interests

The authors have no competing interests, financial or non-financial.

## Authors’ contributions

KM had overall responsibility in the design of first phase of the study (the qualitative phase) across all sites, which included training the researchers, collaborating with researchers on the research protocol, analyzing the data, and drafting this manuscript. HL worked with KM on the analysis for this manuscript, and also helped KM in the writing of the manuscript. The other co-authors helped in carrying out the data collection and analysis in their own study site, and all contributed in providing feedback and edits for this manuscript. All authors read and approved the final manuscript.

## Pre-publication history

The pre-publication history for this paper can be accessed here:

http://www.biomedcentral.com/1471-2458/14/349/prepub
